# Interactions between prolactin and kisspeptin to control reproduction

**DOI:** 10.1590/2359-3997000000230

**Published:** 2016-11-07

**Authors:** Jose Donato, Renata Frazão

**Affiliations:** 1 Departamento de Fisiologia e Biofísica Instituto de Ciências Biomédicas Universidade de São Paulo São Paulo SP Brasil Departamento de Fisiologia e Biofísica, Instituto de Ciências Biomédicas, Universidade de São Paulo (USP), São Paulo, SP, Brasil; 2 Departamento de Anatomia Instituto de Ciências Biomédicas USP São Paulo SP Brasil Departamento de Anatomia, Instituto de Ciências Biomédicas, USP, São Paulo, SP, Brasil

**Keywords:** Prolactin receptor, pSTAT5, hypothalamus, Kiss1, infertility

## Abstract

Prolactin is best known for its effects of stimulating mammary gland development and lactogenesis. However, prolactin is a pleiotropic hormone that is able to affect several physiological functions, including fertility. Prolactin receptors (PRLRs) are widely expressed in several tissues, including several brain regions and reproductive tract organs. Upon activation, PRLRs may exert prolactin’s functions through several signaling pathways, although the recruitment of the signal transducer and activator of transcription 5 causes most of the known effects of prolactin. Pathological hyperprolactinemia is mainly due to the presence of a prolactinoma or pharmacological effects induced by drugs that interact with the dopamine system. Notably, hyperprolactinemia is a frequent cause of reproductive dysfunction and may lead to infertility in males and females. Recently, several studies have indicated that prolactin may modulate the reproductive axis by acting on specific populations of hypothalamic neurons that express the *Kiss1 *gene. The *Kiss1* gene encodes neuropeptides known as kisspeptins, which are powerful activators of gonadotropin-releasing hormone neurons. In the present review, we will summarize the current knowledge about prolactin’s actions on reproduction. Among other aspects, we will discuss whether the interaction between prolactin and the *Kiss1-*expressing neurons can affect reproduction and how kisspeptins may become a novel therapeutic approach to treat prolactin-induced infertility.

## INTRODUCTION

Prolactin is a protein hormone produced and secreted by the anterior pituitary gland. Sparse evidence, at least in rodents, suggests that prolactin may also be locally produced in some brain areas, but the physiological importance of this production is unknown (1). Prolactin secretion is controlled by hypothalamic endocrine neurons, especially the tuberoinfundibular dopamine (TIDA) neurons located in the arcuate nucleus of the hypothalamus (ARH). These neurons secrete dopamine into the hypophyseal portal system, which leads to the activation of dopamine D2 receptors in pituitary lactotrophs, causing suppression of prolactin gene expression and prolactin secretion (1). Prolactin is transported by the circulatory system and acts on target cells via specific receptors located on the plasma membrane (1,2). Serum prolactin crosses the blood-brain barrier by a PRLRs-independent mechanism (3). The function of PRLRs in TIDA neurons is to allow these cells to sense circulating prolactin levels and consequently regulate pituitary prolactin secretion through negative feedback mechanisms. Several other cell populations also express PRLRs, including different brain regions, as well as the bone, adipose tissue, gut, skin, immune system and reproductive tract (2,4-9).

Clinical evidence indicates that hyperprolactinemia is a frequent cause of reproductive dysfunction and may lead to infertility in males and females (10-13). Pathological hyperprolactinemia is mainly caused by the presence of a prolactinoma or is due to pharmacological effects induced by drugs that interact with the dopamine system. Loss-of-function mutations in the gene that encodes the PRLRs can also be a rare cause of hyperprolactinemia (11). The objective of the present review is to summarize and discuss recent advances in the discovery of possible mechanisms linking prolactin signaling and the control of reproduction, especially regarding the role of kisspeptins as novel potential targets to treat prolactin-induced infertility.

## PROLACTIN SIGNALING

Multiple isoforms of membrane-bound PRLRs have been identified, differing in the length and composition of their cytoplasmic tail. In rats, for example, the following three major PRLRs isoforms were identified: short (291 amino acids), intermediate (393 amino acids), and long (591 amino acids). In mice, one long and three short isoforms of the PRLRs have been described (2). After PRLRs activation, different intracellular signaling pathways can be recruited to induce prolactin biological effects. The receptor activation results in a rapid phosphorylation of Janus kinase 2 (JAK2), which is constitutively associated with the intracellular domain of PRLRs. Activation of JAK2 leads to the phosphorylation of tyrosine residues. Phosphotyrosines serve as binding sites for transducer molecules, such as the signal transducer and activator of transcription (STAT) protein family. Three members of this family are recognized as transducer molecules of PRLRs, including STAT1, STAT3 and STAT5a/b. STAT5, earlier known as mammary gland factor, is recognized as the most important transducer of the PRLRs. STAT proteins become phosphorylated by the PRLR/JAK2 complex and form hetero- or homodimers through its phosphotyrosine residues with another phosphorylated STAT molecule. STAT dimers then translocate to the nucleus, where genomic effects on target genes can occur. The phosphotyrosine residues of the activated PRLR may also serve as a docking site for others adapter proteins which can lead to the activation of different signaling pathways, such as the mitogen-activated protein kinase (MAPK) cascade or the phosphatidylinositol 3-kinase (PI3K) cascade (1,2). In addition, it has been demonstrated that PRLRs activation is also involved in rapid acute effects that lead to changes in membrane excitability. For example, prolactin acutely induces rapid effects on the membrane excitability of neurons (14-18). Such effects occur because PRLRs activation can activate fast-acting signaling mechanisms, such as the PI3K pathway, tyrosine kinase-dependent K^+^ channels or the production of intracellular messengers that open voltage-independent Ca^2+^ channels, which in turn allows for ionic changes across the cell membrane (1,15). One of the final mechanisms induced by PRLRs activation is protein synthesis that can in turn desensitize the receptor itself. The JAK/STAT complex can be inhibited by suppressors of cytokine signaling (SOCS) proteins, which inhibit JAK kinases and compete with STAT for docking sites on PRLRs. These proteins include SOCS1, SOCS3 and cytokine-inducible SH2-containing protein (CIS) (1).

## PROLACTIN-MEDIATED REPRODUCTIVE FUNCTIONS IN PERIPHERAL ORGANS

Prolactin is best known for its role in mammopoiesis and lactogenesis. Mammary gland development includes the formation of a branched ductal system that is decorated with terminal and lateral lobules in wild-type virgin adult mice. Following pregnancy and in response to prolactin production, the alveolar development of the mammary gland is greatly amplified. Several studies have provided consistent evidence that such progression is directly modulated by the recruitment of STAT5 proteins upon PRLRs activation. In PRL^-/-^ or PRLR^-/-^ female mice, terminal end buds form during puberty and the ductal tree grows normally. However, in adult PRL^-/- ^mice, the mammary gland ductal system grows into an extended branching network that is devoid of both terminal and lateral lobulations (19). The differentiation of ductal elements also occurs in the global STAT5a, STAT5b and double knockout STAT5a/b female mice; however, development of terminal buds occurs to a lesser extent as compared to wild-type mice (20,21). Deficiencies in mammary gland development were even observed in non-lactating heterozygous PRLRs knockout female mice, indicating that such development is dependent upon prolactin signaling (22). Of note, mammary gland development upon pregnancy could not be observed in PRLR^-/- ^female mice, as well as in global double STAT5a/b knockout females due to their infertility. The total body of research has led to the determination that STAT5 is the principal transcription factor mediating mammopoiesis and lactogenesis. Global STAT5a knockout mice fail to lactate due to incomplete mammary gland development, even after maximal stimulation of prolactin secretion induced by suckling. Conversely, mammary gland development occurs in a relatively normal manner in STAT5b knockout mice (20,21). In addition, the specific deletion of the *Stat5* locus only from the mammary epithelium, using *Cre-loxp*-mediated recombination, determined that this protein is essential not only for pregnancy-mediated cell proliferation/differentiation but also for the survival of mammary epithelium and maintenance of differentiation (23).

The effects of prolactin on fertility have been well-characterized using knockout mouse models, indicating that reproduction is clearly dependent upon the signaling of this hormone, at least in rodents. Both short and long isoforms of PRLRs have been described to be expressed in the granulosa, interstitial and luteal cells of the ovaries, and the endometrium, myometrium and decidua in the uterus (24-26). Estradiol is the main ovarian hormone that stimulates prolactin secretion. Estradiol acts at the pituitary level to modulate prolactin gene expression and at the hypothalamus to modulate the activity of neurons known to be prolactin responsive (1,9). Depending on the hormonal milieu, PRLRs expression in several tissues (*e.g*., the ovaries, uterus and hypothalamus) may change along the estrous cycle or during pregnancy and lactation (24,25). In the ovaries, prolactin acts in concert with gonadotropins to stimulate progesterone production by luteal cells and to induce the increase in progesterone receptor expression in the uterus. Progesterone produced by the ovaries is essential for implantation of the fertilized ovum, maintenance of pregnancy and the inhibition of ovulation (2). Prolactin signaling disruption leads to reproductive deficits in mice. For example, PRL^-/- ^or PRLR^-/- ^female mice are infertile (19,22). Adult PRL^-/- ^female mice have irregular estrous cycles, with multiple days of proestrus or estrus. These mutants fail to become pregnant, despite no obvious defects in ovarian histology (19). Similarly, PRLR^-/- ^female mice are infertile, despite regularly mating every 3-4 days, in comparison to wild-type animals that mated every 12 days. The study of preimplantation development of embryos demonstrated that most of the fertilized eggs failed to develop correctly in PRLR^-/- ^mothers, although the embryos that failed to develop were capable of normal development when transferred to wild-type mothers. In addition, it was found that the uterus of a PRLR^-/- ^female was not able to accept the implantation of wild-type blastocysts, indicating that the lack of PRLRs made the uterus refractory to implantation (22). Despite the reproductive defects exhibited by mutant females, PRL^-/-^ male mice are fully fertile, and most PRLR^-/- ^males are fertile, which demonstrates that the infertility in knockout females is caused by the lack of prolactin’s luteinizing effects (19,22). However, it is worth mentioning that prolactin has this particular function only in rats and mice, but not in all mammals. Double STAT5a/b knockout female mice, but not solely STAT5a or STAT5b knockouts, are infertile as well (20,21). STAT5a/b knockout mice ovaries exhibit either few or no corpora lutea, which was determined to be the main cause of their infertility (21).

## THE HYPOTHALAMUS AS A TARGET OF PROLACTIN TO MODULATE SEVERAL BIOLOGICAL FUNCTIONS

**Figure 1:**
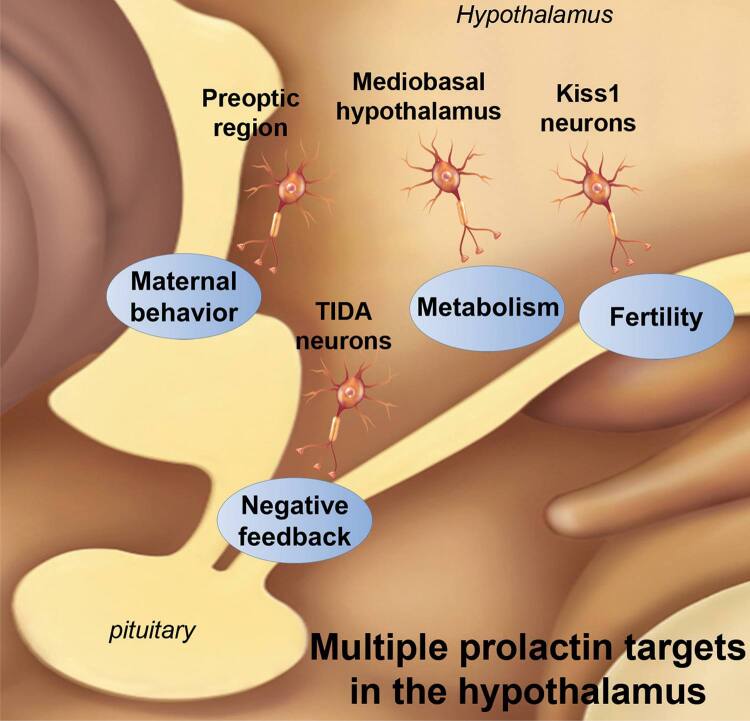
Scheme that summarizes different biological functions regulated by prolactin through its action on defined hypothalamic neuronal populations.

Prolactin-responsive cells are densely distributed in the central nervous system, especially in the hypothalamus (9,27,28). Several biological functions are regulated by prolactin through its action on defined hypothalamic neuronal populations, including the regulation of prolactin secretion through negative feedback, the expression of maternal behaviors and the modulation of energy balance and the reproductive axis (Figure 1). The best known hypothalamic circuitry involving prolactin effects is composed of TIDA neurons that act as a synchronous network to release dopamine and control prolactin secretion (1,15-17). TIDA neurons are directly responsive to prolactin as demonstrated by the induction of STAT5 phosphorylation (pSTAT5) after an acute prolactin stimulus and a direct postsynaptic depolarization of cell membranes, which stimulates dopamine secretion (15-17). However, during lactation, dopamine secretion is suppressed to allow for physiological hyperprolactinemia. Because TIDA neurons remain electrically responsive to prolactin during lactation, the significant decrease in tyrosine hydroxylase phosphorylation is the best-known mechanism so far that is responsible for the suppression of dopamine secretion during this period (17). In addition, prolactin is also known as an important factor that mediates adaptive responses related to maternal behaviors. In this case, the effects of prolactin on maternal care depend on neurons distributed in the preoptic area (29). In rodents, maternal care can be analyzed by evaluating the latency of the animal to exhibit behaviors, such as retrieving pups to the nest, grouping them and crouching over them for a set amount of time. Previous studies performed bilateral infusions of prolactin into the preoptic region, which resulted in a pronounced stimulation of maternal behavior (30). Animals infused with prolactin directly into the medial preoptic area (MPO) retrieved the pups, crouched over them and displayed full maternal behavior significantly faster than respective controls (30). In contrast, overall full maternal response was impaired in animals given bilateral infusions of a PRLRs antagonist into the MPO (31). Interestingly, while PRLR^-/-^ female mice showed a complete disruption of maternal behavior (22,32), brain-specific STAT5a/b knockout mice showed no postpartum maternal behavior deficits, demonstrating that STAT5 signaling is not required for the expression of maternal care. Consequently, other signaling pathways recruited by PRLRs activation may be necessary to modulate maternal behavior. In fact, we have previously demonstrated that fast STAT5-independent signaling pathways are acutely recruited by prolactin to modulate the membrane excitability of neurons located in the MPO (14).

Prolactin may also regulate food intake and other metabolic aspects. Hyperprolactinemia is frequently associated with metabolic imbalances, such as obesity and diabetes mellitus (33). Accordingly, PRLR^-/-^ mice show a lean phenotype (34). Several studies also indicate that prolactin or placental lactogens contribute to the metabolic changes typically observed during pregnancy, such as the increase in food intake and adiposity. These studies showed that central prolactin infusions induce a leptin resistance state that changes the metabolism towards a positive energy balance (35,36). Indeed, several populations of leptin receptor-expressing neurons in mice are directly responsive to prolactin (37). Furthermore, inactivation of the *Socs3* gene in leptin receptor-expressing cells improves leptin sensitivity in pregnant mice and mitigates major gestational metabolic adaptions (38). Altogether, these findings suggest that changes in prolactin levels during pregnancy lead to leptin resistance, which, in turn, is responsible for orchestrating many metabolic adaptations commonly observed in pregnant animals. Thus, these studies indicate that prolactin may centrally modulate energy balance in specific situations.

## INTERACTION BETWEEN PROLACTIN AND THE KISSPEPTIN SYSTEM

**Figure 2:**
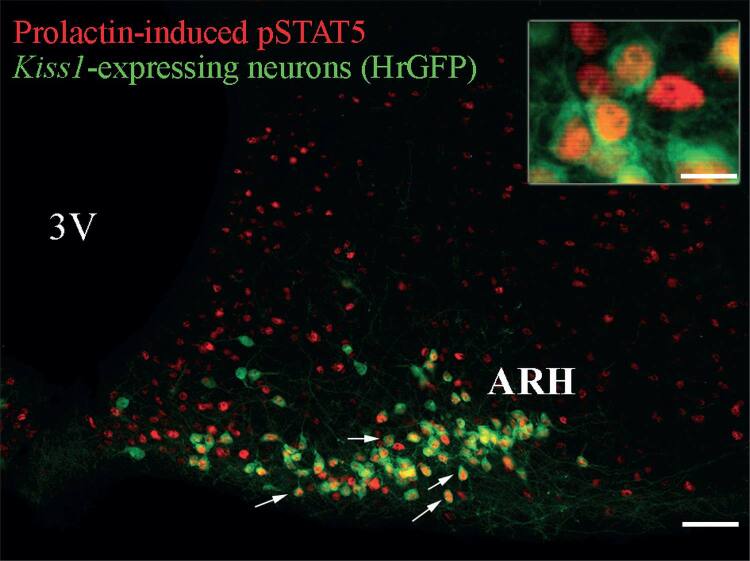
Prolactin-responsive *Kiss1-*expressing neurons in the mouse arcuate nucleus. Fluorescent photomicrographs of hypothalamic sections showing the expression of humanized Renilla green fluorescent protein (hrGFP) which is expressed under the transcriptional control of the *Kiss1* gene and prolactin-induced pSTAT5 immunoreactivity of a female mouse in diestrus. Details about this mouse model can be found in previous publications (Cravo and cols., 2013). Mice received a single i.p. injection of ovine prolactin (10 µg/g) before perfusion. Arrows illustrate examples of prolactin-responsive *Kiss1*-expressing neurons. The *inset *represents a higher magnification of dual-labeled neurons in the ARH. Abbreviations: 3V, third ventricle. Scale bar: photomicrograph = 100 µm; inset = 23 µm.

Hyperprolactinemia frequently causes disruption of gonadotropin-releasing hormone (GnRH), luteinizing hormone (LH) and follicle stimulating hormone (FSH) secretion, and may lead to hypogonadism and infertility in humans and animal models (12,39-41). The most common symptoms of hyperprolactinemia in women are galactorrhea, amenorrhea and infertility. In men, the most frequent symptoms are typically secondary to a sellar mass effect, such as headaches and visual field defects due to the pituitary enlargement (40,41). A small percentage of male patients present with symptoms such galactorrhea, loss of libido, erectile dysfunction, changes in sperm quality and infertility (12,40). To better understand how hyperprolactinemia affects reproduction, several studies investigated possible prolactin-target neurons that may modulate the reproductive axis. Although GnRH neurons were thought to be potential candidates to be directly regulated by prolactin, it has been demonstrated that only a very small percentage of GnRH neurons express the PRLRs or prolactin-induced pSTAT5. In addition, membrane excitability of GnRH neurons is not acutely modulated by prolactin (16,42), suggesting that other neuronal populations are probably responsible for the prolactin-mediated effects on gonadotropin secretion. Recently, studies provided new evidence that prolactin may modulate the reproductive axis by acting on a specific population of hypothalamic neurons that express the *Kiss1* gene (4-8,43). The* Kiss1* gene encodes neuropeptides, known as kisspeptins, that are critically involved in reproduction. Loss-of-function mutations in the genes encoding kisspeptins or the kisspeptin receptor (*KISS1R*, also known as *GPR54*) leads to the disruption of puberty and infertility in both humans and animal models (44-46). Conversely, a *KISSR*-activating mutation leads to precocious puberty in humans (47). *Kiss1*-expressing neurons exhibit a very defined distribution in the brain. These neurons are mainly located in the anteroventral periventricular nucleus (AVPV), the rostral periventricular nucleus (PeN) and the ARH of the hypothalamus in rodents (48). Of note, some research groups denominate AVPV and PeN neurons together as the rostral periventricular area of the third ventricle (RP3V) in rodents (4,42). The confirmation that *Kiss1*-expressing neurons are directly modulated by prolactin levels was provided by the demonstration that most of *Kiss1*-expressing neurons co-express the PRLRs (6,7). Additionally, these receptors are functional because an acute prolactin stimulus can induce pSTAT5 in *Kiss1*-expressing neurons (detailed information can be found on Table 1) (4,5,8). Similar results were obtained by our group using a transgenic mouse model that allows the visualization of *Kiss1*-expressing neurons through a reporter protein. Approximately 80% of *Kiss1*-expressing neurons in the ARH express pSTAT5 after acute intraperitoneal (i.p.) prolactin administration in female mice in diestrus (Figure 2). The expression of PRLRs and the induction of pSTAT5 by prolactin together indicate that prolactin may regulate the activity of *Kiss1*-expressing neurons and kisspeptin secretion. More evidence that the interaction between prolactin and kisspeptin system causes significant impact to the hypothalamic-pituitary-gonad axis was provided by studies that evaluated the consequences of prolactin infusion on *Kiss1 *mRNA expression in animal models (Table 1). Systemic or intracerebroventricular (icv) prolactin infusion suppresses hypothalamic *Kiss1* expression leading to a reduction in plasma LH levels (4,5,43).

**Table 1:** Summary of the studies that investigated the interaction between prolactin and kisspeptin

Reference	Species	Comments	
**Presence of prolactin receptors in *Kiss1*-expressing neurons**			

Kokay and cols., 2011	Rat	86% of *Kiss1-*expressing neurons in the AVPV co-express PRLRs mRNA in OVX+E2 treated females 79% and 45% of *Kiss1*-expressing neurons in the ARH of OVX or OVX+E treated animals, respectively, co-express PRLRs mRNA	
Li and cols., 2011	Sheep	60% of ARH kisspeptin immunoreactive neurons co-express PRLRs in OVX females	
	Human	Not described	

**Evidence of responsiveness to prolactin**			

Brown and cols., 2014	Mouse	65% of kisspeptin immunoreactive neurons in the AVPV and 35% of kisspeptin neurons in the PeN express prolactin-induced pSTAT5 in mice in diestrus	
Araujo-Lopes and cols., 2014	Rat	70-80% of ARH kisspeptin immunoreactive neurons express pSTAT5 after prolactin icv infusion (0.5 µg/2 µL) in virgin OVX or lactating animals	
Sjoeholm and cols., 2011	Rat	65-75% of ARH kisspeptin neurons of primiparous rats co-express pSTAT5 after icv prolactin infusion (2.5 or 100 ng/rat)	
Li and cols., 2011	Sheep	No co-expression between *Kiss1 *mRNA and prolactin-induced pSTAT5 (icv, 20 µg/hr/1 week) in the ARH of OVX+E2 females	
	Human	Not described	

**Effects of prolactin on *Kiss1* expression**			

Sonigo and cols., 2012	Mouse	Chronic infusion of prolactin (7 μg/24 hr/28 days) significantly decreases hypothalamic* Kiss1 *mRNA and kisspeptin immunoreactivity in the AVPV and ARH	
Brown and cols., 2014	Mouse	3 sc doses of prolactin (100 µg/200 µg) cause a suppression of *Kiss1* mRNA in the ARH of OVX mice	
Araujo-Lopes and cols., 2014	Rat	Icv (4 µg/µL) or sc (0.5 mg/0.2 mL) prolactin injection causes a significant reduction of *Kiss1 *mRNA in the ARH of OVX rats	
Li and cols., 2011	Sheep	Icv prolactin infusion (20 µg/hr/1 week) does not significantly change *Kiss1 *mRNA expression in the ARH of OVX+E2 females	
	Human	Not described	

**Effects of kisspeptin on prolactin secretion**			

Szawka and cols., 2010	Rat/cell culture	Kisspeptin-10 icv infusion (3 nmol) increases serum prolactin release in OVX+E2 and proestrus females, but had no effect in OVX females, diestrus females or males Kisspeptin-10 does not alter prolactin secretion in anterior pituitary cell culture	
Hashizume and cols., 2010	Goat	Intravenous administration of kisspeptin-10 (5 mg/kg) does not alter basal serum prolactin levels	
Kadokawa and cols., 2008	Bovine/cell culture	Kisspeptin-10 (1 µM or 10 µM) increases media prolactin concentration of anterior pituitary cells, extracted from 8-month-old castrated male calves	
Ramaswamy and cols., 2009	Monkey	Intravenous infusion of kisspeptin-10 (10 or 30 μg) induces no change in prolactin serum concentration in male *Macaca mulatta*	
Jayasena and cols., 2014	Human	Acute or twice-daily for 1 week sc administration of kisspeptin-54 (6.4 nmol/kg) induced no effect on serum prolactin levels in healthy women	

Interestingly, not only may prolactin affect the activity of *Kiss1*-expressing neurons, but kisspeptins possibly control prolactin secretion as well. Administration of kisspeptin-10 to culture media prepared with anterior pituitary cells extracted from 8-month-old castrated male calves increased prolactin secretion (49). However, several other studies were unable to demonstrate such effect (Table 1) (50-53). In humans, for example, acute or a week of twice-daily subcutaneous (sc) administrations of kisspeptin-54, at a dose known to stimulate gonadotropin secretion, induced no effect on serum prolactin levels in healthy women (53). In fact, the ability of kisspeptin to induce prolactin secretion seems to be directly related to circulating estrogen levels, as demonstrated in rodents. Icv infusion of kisspeptin-10 increases serum prolactin levels only in ovariectomized E2-primed (OVX+E2) or proestrus rats, but had no effect in ovariectomized (OVX) females, diestrus females or male rats (52). Additionally, Ribeiro and cols. (54) demonstrated that kisspeptins increase prolactin secretion through inhibition of TIDA neurons in an estrogen-dependent manner, not only in female rats but also in males. The demonstration, at least in rodents, that kisspeptin administration may induce prolactin secretion through TIDA neurons inhibition suggests that *Kiss1-*expressing neurons may also be part of the feedback circuitry responsible for modulating prolactin secretion.

## MAY KISSPEPTIN BE USED AS A NOVEL THERAPEUTIC APPROACH TO TREAT PROLACTIN-INDUCED INFERTILITY?

Because kisspeptins are known as the most important activators of GnRH neurons (55) and they are able to cause a powerful stimulation in LH and FSH secretion in both humans and animal models (48,56-58), kisspeptin administration may have potential therapeutic effects to treat infertility. The first evidence of this effect was provided by a clinical trial in which women with functional hypothalamic amenorrhea were subjected to a protocol of twice-daily sc kisspeptin-54 administration for 2 weeks. After the first kisspeptin-54 injection, women with functional hypothalamic amenorrhea showed a rapid and marked increase in plasma gonadotropins and estradiol levels, in comparison to the vehicle group. However, 2 weeks of kisspeptin-54 treatment led to receptor desensitization and no further significant effect on gonadotropins secretion was observed (58). In fact, the treatment of women with hypothalamic amenorrhea was more effective when kisspeptin-54 was administered sc twice-weekly over a prolonged period. This protocol elevated the levels of reproductive hormones even after 8 weeks of treatment (59). Additionally, the effects of kisspeptins administration on egg maturation in women undergoing in vitro fertilization therapy have also been tested. A single sc injection of kisspeptin-54 was able to trigger egg maturation sufficiently to result in fertilization, embryo implantation, and successful live birth in women with subfertility. Of note, the efficacy rate of kisspeptin treatment was similar to that obtained by conventional therapy (60).

Kisspeptin administration can also restore gonadotropin secretion and ovarian cyclicity in a prolactin-induced infertility model in mice (43). Hyperprolactinemia was induced by a chronic sc infusion of prolactin. While control animals displayed regular estrous cycles, hyperprolactinemic female mice showed disruption of their cycles. Remarkably, daily i.p. injections of kisspeptin-10 recovered estrous cyclicity and ovulation rate even in hyperprolactinemic mice (43). Additionally, Sonigo and cols., (43) tested whether the suppression of GnRH release induced by hyperprolactinemia could be reversed by kisspeptin treatment. To demonstrate the effects of kisspeptins on GnRH secretion, medial basal hypothalamus explants obtained from female mice were treated in a culture medium. As expected, GnRH release into the medium was significantly inhibited after exposure to prolactin. Notably, co-treatment with kisspeptin-10 was able to restore GnRH secretion, demonstrating that prolactin inhibitory action on gonadotropin secretion is mediated by changes in kisspeptin secretion (43). Therefore, the reduction of *Kiss1 *gene expression is now believed to be the primary cause of the suppression of gonadotropin secretion during hyperprolactinemia. Nevertheless, whether kisspeptin may be used as a novel therapeutic approach to treat prolactin-induced infertility in humans still requires further investigation.

In conclusion, recent evidence indicates that *Kiss1-*expressing neurons are important mediators of prolactin’s effects on reproduction. Prolactin acts directly on *Kiss1-*expressing neurons and induces suppression of *Kiss1* mRNA expression and kisspeptin secretion, leading to a lower activation of GnRH and gonadotropins secretion. Therefore, hyperprolactinemia-induced infertility can possibly be treated with kisspeptin replacement. Furthermore, kisspeptins seem to contribute to the control of prolactin secretion, which highlights a putative bidirectional interaction between prolactin and the kisspeptin system.
